# Quantitative Structure–Reactivity Relationships for Synthesis Planning: The Benzhydrylium Case

**DOI:** 10.1021/acs.jpca.3c07289

**Published:** 2023-12-19

**Authors:** Maike Eckhoff, Johannes V. Diedrich, Maike Mücke, Jonny Proppe

**Affiliations:** † Institute of Physical and Theoretical Chemistry, TU Braunschweig, Braunschweig 38106, Germany; ‡ Institute of Physical Chemistry, University of Göttingen, Göttingen 37077, Germany

## Abstract

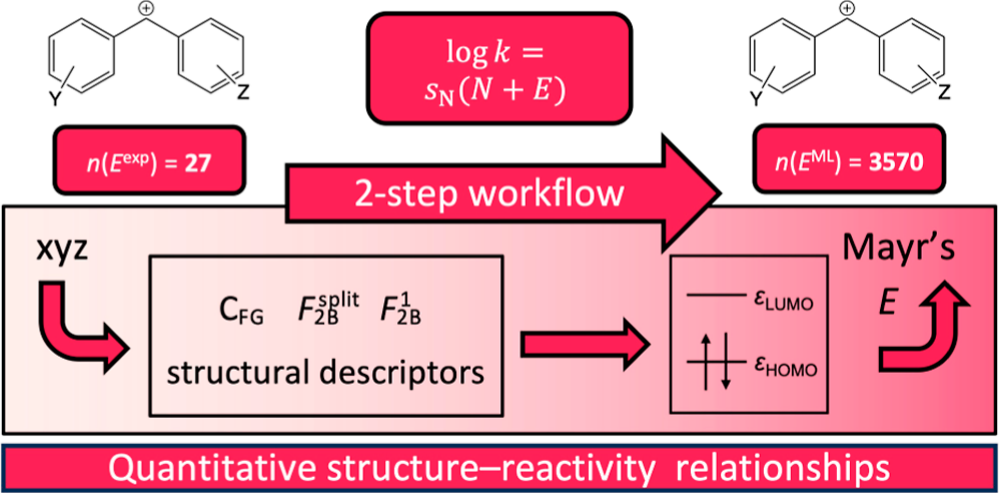

Selective and feasible reactions are among the top targets in synthesis planning. Mayr’s approach to quantifying chemical reactivity has greatly facilitated the planning process, but reactivity parameters for new compounds require time-consuming experiments. In the past decade, data-driven modeling has been gaining momentum in the field, as it shows promise in terms of efficient reactivity prediction. However, state-of-the-art models use quantum chemical data as input, which prevent access to real-time planning in organic synthesis. Here, we present a novel data-driven workflow for predicting reactivity parameters of molecules that takes only structural information as input, enabling *de facto* real-time reactivity predictions. We use the well-understood chemical space of benzhydrylium ions as an example to demonstrate the functionality of our approach and the performance of the resulting quantitative structure–reactivity relationships (QSRRs). Our results suggest that it is straightforward to build low-cost QSRR models that are accurate, interpretable, and transferable to unexplored systems within a given scope of application. Moreover, our QSRR approach suggests that Hammett σ parameters are only approximately additive.

## Introduction

The ability to predict whether two compounds will react and, if so, how fast, is essential for synthesis planning. The estimation of relative rates and hence selectivity is equally vital as they determine the likelihood of unwanted side reactions taking place during the synthesis process. Knowledge of these fundamental variables is rooted in the reactivity of the molecules involved. Quantitative reactivity scales,^1^ building on the “golden” decades of physical organic chemistry,^2^ enable chemists to make informed decisions about which reactions to pursue, thereby saving time, resources, and effort in the laboratory. As important and helpful these scales are, their experimental determination is rather time-consuming.

By leveraging advances in hardware, algorithms, and data science, a plethora of new efficient tools for planning organic syntheses has become available in the past decade.^3−11^ These new techniques can rapidly provide valuable insights into the reactivity of molecules, enabling chemists to make informed decisions during routine synthesis design. This approach has the potential to significantly accelerate the discovery and development of novel compounds that serve as drugs or building blocks of functional materials.

Our group currently explores the suitability of quantitative structure–reactivity relationships (QSRRs) for accelerating reactivity predictions and, hence, synthesis planning.^12−14^ We aspire to build an interactive platform on which users can query arbitrary organic compounds and receive *instant* feedback, including site-specific reactivity information and uncertainty estimates^15^ to ensure reliability and practical benefit. With the ability to assess reactivity in real time, chemists can efficiently evaluate a vast number of compounds and potential reactions and choose the most promising ones for further investigation.

**Figure 1:**
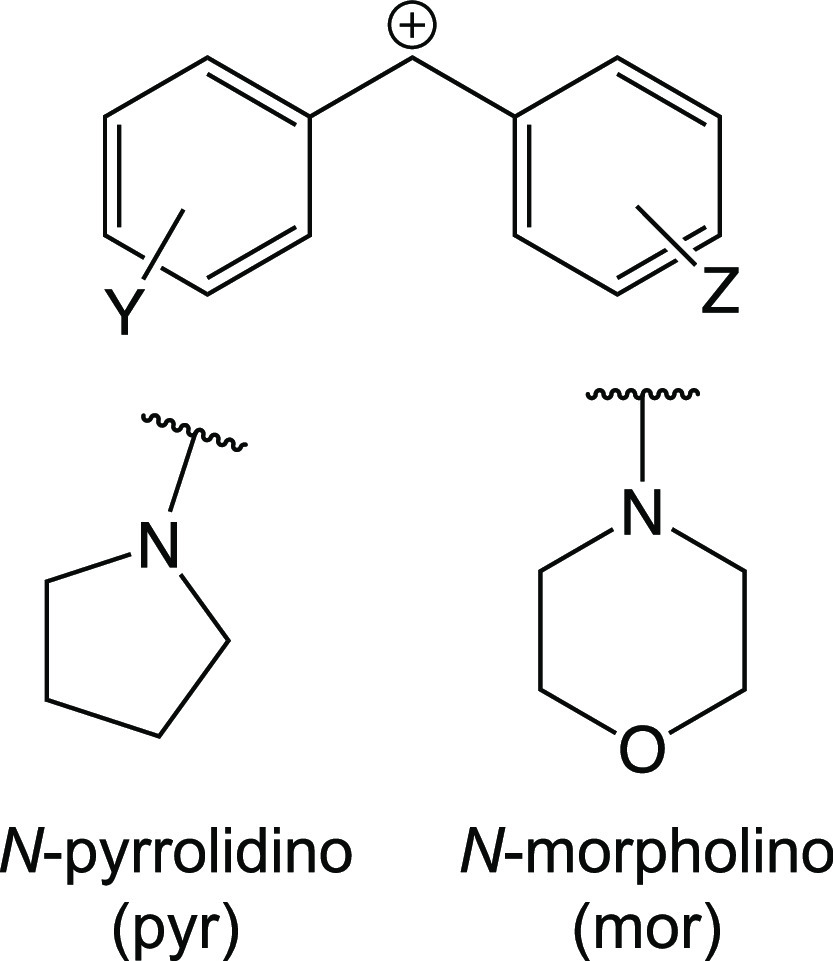
Top: The benzhydrylium scaffold has substituents *Y* and *Z*, which are specified in Table 1. Bottom: Lewis structures of the *N*-pyrrolidino and *N*-morpholino substituents.

Here, we present a proof of principle using the chemical space of benzhydrylium ions (Figure 1 and Table 1) as an example. The benzhydrylium ion and its derivatives tell a success story in terms of quantifying chemical reactivity. Driven by the attempt to systematize the use of carbocations in organic synthesis, Mayr and co-workers studied reactions of olefins with benzhydrylium ions.^16,17^ Mayr’s team was astonished when they found that the relative reactivity of most alkenes is independent of the reactivity of the benzhydrylium ion they react with.^18,19^ Eventually, Mayr and Patz proposed a simple expression containing only three empirical parameters to compute the rate constant of polar bimolecular reactions in solution^20^
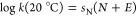
1

**Table 1:** Benzhydrylium Ion Derivatives of Mayr’s Database Considered in This Work^a^

ID	*Y*	*Z*	*E*(1)	ID	*Y*	*Z*	*E*(1)
**1**	*p*-pyr	*Y*	–7.69	**15**	*p*-Me	H	4.43
**2**	*p*-dma	*Y*	–7.02	**16**	*p*-F	*Y*	5.01
**3**	*p*-mpa	*Y*	–5.89	**17**	*p*-F	H	5.20
**4**	*p*-mor	*Y*	–5.53	**18**	3-F, *p*-Me	*Y*	5.24
**5**	*p*-dpa	*Y*	–4.72	**19**	H	*Y*	5.47
**6**	*p*-mfa	*Y*	–3.85	**20**	*p*-Cl	*Y*	5.48
**7**	*p*-pfa	*Y*	–3.14	**21**	3-F	H	6.23
**8**	*p*-OMe	*Y*	0.00	**22**	*p*-(CF_3_)	H	6.70
**9**	*p*-OMe	*p*-OPh	0.61	**23**	3,5-F_2_	H	6.74
**10**	*p*-OMe	*p*-Me	1.48	**24**	3-F	*Y*	6.87
**11**	*p*-OMe	H	2.11	**25**	3,5-F_2_	3-F	7.52
**12**	*p*-OPh	*p*-Me	2.16	**26**	*p*-(CF_3_)	*Y*	7.96
**13**	*p*-OPh	H	2.90	**27**	3,5-F_2_	*Y*	8.02
**14**	*p*-Me	*Y*	3.63				

a Substituents *Y* and *Z* (*cf.*Figure 1) as well as electrophilicity parameters *E*(1) are listed. See Table S7 for reference electrophile names. The following abbreviations are used: *p*-dma, 4-(dimethylamino)phenyl; *p*-dpa, 4-(diphenylamino)phenyl; *p*-mpa, 4-(methylphenylamino)phenyl; *p*-mfa, 4-(methyl(trifluoroethyl)amino)phenyl; *p*-pfa, 4-(phenyl(trifluoroethyl)amino)phenyl; *p*-pyr, 4-(*N*-pyrrolidino); *p*-mor, 4-(*N*-morpholino).

Here, *E*, *N*, and *s*_N_ represent electrophilicity, nucleophilicity, and a nucleophile-specific sensitivity parameter, respectively. As they proceeded, Mayr and his team found that the Mayr–Patz equation (eq 1) is also valid for many other classes of nucleophiles and electrophiles. To date, reactivity parameters have been determined for 352 electrophiles (*E*) and 1281 nucleophiles (*N*, *s*_N_), which can be accessed via *Mayr’s Database of Reactivity Parameters*.^21,22^ A brief explanation of how these parameters are determined experimentally^23,24^ is given in boxes 1 and 2 of ref (14).

Attempts have been made to determine reactivity parameters by thermochemical calculations based on density functional theory (DFT). However, they have not yet prevailed over the experimental approach also because of accuracy issues. In a recent uncertainty quantification study,^15^ we confirmed that the average accuracy of experimental rate constants corresponding to reactions of olefins with benzhydrylium ions is higher—deviation in *k* below 1 order of magnitude—than that achievable with standard DFT calculations. Even high-performing functionals result in average barrier height errors of at least 2 kcal mol^–1^,^25^ translating to a deviation in *k* of 1 to 2 orders of magnitude at 20 °C assuming validity of the Eyring equation.^26^ Ultimately, DFT is not suitable for the efficient prediction of the reactivity parameters. This is one reason why data-driven or machine-learning (ML) algorithms have gained much attention in this context as they are capable of yielding fast predictions by interpolating between available data.^14^

In supervised ML, relationships between descriptors (input variables) and targets (output variables) are learned by means of regression (continuous target) or classification (discrete target). Aside from the expensive acquisition of targets (*i.e.*, experimental reactivity parameters), the generation of descriptors can constitute a critical bottleneck in the ML workflow. For instance, previous data-driven studies have mostly relied on quantum molecular properties (QMPs) as descriptors,^27−37^ meaning that each prediction is preceded by quantum chemical (mainly DFT) calculations, which occupy almost 100% of the overall prediction time. This applies less to semiempirical electronic-structure methods, as recently applied in a related context,^38^ which can much more efficiently generate QMPs and other electronic descriptors.

While QMPs are among the most informative descriptors,^33^ we target descriptors that can be both generated *quickly* and *interpreted* intuitively. For this purpose, we focus on *structural* descriptors in this work. Structural descriptors are direct representations of the connectivity/graph or the three-dimensional structure of a molecule.^39^ There are two principal types of structural descriptors: General (application-agnostic) descriptors, which are applicable to a broad range of structure classes but rather difficult to interpret. On the other hand, application-specific descriptors are rather simple to interpret but not generalizable to cases outside the domain of application. We are particularly interested in the latter type of descriptors but will investigate the merits and drawbacks of both types.

**Figure 2:**
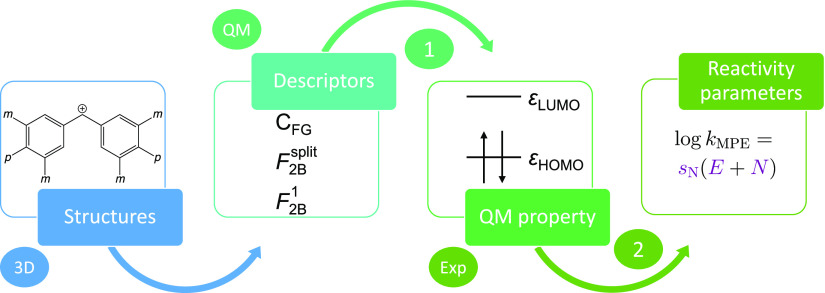
Schematic description of our two-step workflow for predicting reactivity parameters from molecular structures. In step 2 of the workflow, the relationship between a suitable quantum molecular (QM) property and the reactivity parameter of interest is learned. Since QM properties require expensive quantum chemical calculations, we seek to replace their calculation in step 1 of the workflow. For this purpose, the three-dimensional (3D) molecular structures are initially transformed into machine-learnable descriptors before the relationships between these descriptors and the QM property are learned.

In general, structural descriptors are much higher in dimensionality than QMPs. As a rule of thumb, the greater the dimensionality of a descriptor, the more data are needed to uncover the underlying QSRR. However, the number of reactivity parameters in Mayr’s database is limited. Therefore, we hypothesize that the structural descriptors examined here are too high-dimensional to be directly linked to the relatively small number of available reactivity parameters. To meet this challenge, we propose a two-step workflow for building QSRR models from structural descriptors (Figure 2).

Assume a set of *K* available reactivity parameters that is too small to build an accurate QSRR model based on a high-dimensional structural descriptor. Further, assume that a set of *L* ≫ *K* reactivity parameters would be necessary to achieve the desired accuracy. Then, if we could identify a less expensive surrogate quantity that correlates well with the reactivity parameter of interest, it would be possible to build such a two-step QSRR model. In this work, we propose QMPs to serve as surrogate quantities. The use of QMPs may seem like a contradiction to the goal of avoiding them, as stated above. However, given a set of *M* molecules of interest, only *L* ≪ *M* of which are equipped with QMPs, we have avoided (multiples of) *M* – *L* quantum chemical calculations, leading to substantial computational savings.

Summarizing: Reactivity parameters for *M* compounds are requested. In step 1, high-dimensional structural descriptors are linked with a small number of QMPs. The training set size of step 1 is *L* ≪ *M*. In step 2, the same QMPs are linked to the actual reactivity parameters. The training set size of step 2 is *K* ≪ *L*.

Step 1 is based on Gaussian process regression (GPR),^40−42^ a powerful nonlinear ML method that we have found to be particularly effective when there are limited data available.^43−45^ Step 2 is based on multivariate linear regression (MLR), a method that has experienced a revival in physical organic chemistry—where MLR models are better known as *linear free energy relationships*(46)—owing to the work by Sigman and co-workers.^47^ The Sigman-type MLR method was adapted by Orlandi *et al.*(35) for predicting and understanding Mayr’s nucleophilicity parameter *N*. To facilitate interpretation of results, MLR is additionally applied in step 1 of our workflow (*i.e.*, GPR for prediction, MLR for understanding).

In this work, we apply the novel two-step QSRR workflow to a data set of *M* = 3570 benzhydrylium ions, for only *K* = 27 of which an electrophilicity parameter *E* is available (see Figure 1 and Table 1). At the same time, these 27 systems cover a wide range of reactivity, −10.04 < *E* < 8.02, spanning almost 20 orders of magnitude. Their electrophilic center, a carbenium ion, can be tuned by distant substituents. As a result, the reactivity of benzhydrylium ions can be dominantly attributed to electronic effects, leading to unambiguous *E* parameters. These electrophiles are therefore particularly well suited for building quantitative nucleophilicity scales for a variety of organic compounds.^1^

After an overview of the data and methods used in this work, the potential of our two-step workflow, in terms of real-time reactivity prediction, is evaluated. In particular, the MLR and GPR models are analyzed with respect to performance (GPR in step 1, MLR in step 2) and interpretability (MLR in both steps). Finally, we analyze the relationship between Mayr’s electrophilicity *E* and (the sum of) Hammett σ parameters.^48^

## Methods

### Data Set

Mayr’s database^21,22^ comprises electrophilicity parameters for 33 benzhydrylium ions, six of which are annulated and therefore removed for the following analysis. Table 1 and Figure 1 show the remaining *K* = 27 electrophiles.

**Figure 3:**
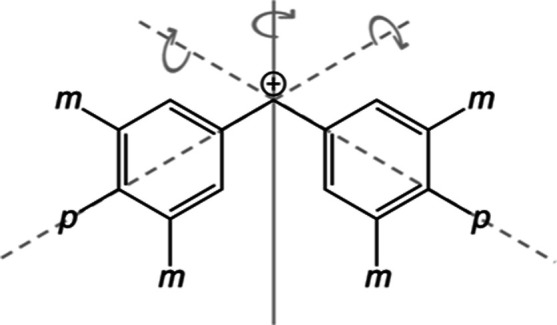
Benzhydrylium scaffold with emphasized *meta* positions *m* and *para* positions *p*. Rotation axes leading to approximately isoenergetic structures are displayed in gray.

For this study, a combinatorial data set of benzhydrylium ion derivatives was generated based on the unsubstituted ion **19**. Its four *meta* (*m*) and two *para* (*p*) positions, which are shown in Figure 3, are suitable substitution sites. By avoiding substitution of the *ortho* positions, the steric situation at the carbenium ion is preserved, and hence, its electrophilicity is predominantly caused by electronic substituent effects. We considered only substituents of benzhydrylium ions available in Mayr’s database (see Table 1), 13 in total: –F, –Cl, –Me, –OMe, –OPh, –dma, –mpa, –dpa, –pyr, –mor, –mfa, –pfa, and –CF_3_. The Lewis structures of the –pyr and –mor groups are shown in Figure 1. Only –F and –Cl were selected as possible *m*-substituents to avoid steric hindrance with the *p*-substituents, while all of the above-mentioned substituents were selected as possible *p*-substituents.

Next, all possible substitution combinations of these functional groups were generated, leading to 3^4^ × 14^2^ = 15876 structures (counting hydrogen as third *m*-substituent and 14^th^*p*-substituent, respectively). If structures could be converted into each other by the *C*_2_ rotation axis, shown as a gray solid line in Figure 3, only one of them was kept. In addition, tests have shown that assuming a symmetry axis passing through the bond between the carbenium ion and the aromatic rings (dashed gray lines in Figure 3) is a reasonable approximation (see Supporting Information Section “Examination of rotational symmetry”). The resulting duplicate molecules were removed, as well. The final data set therefore consists of *M* = 3570 structures, in which the *K* = 27 aforementioned reference structures are present.

### Descriptors

For the data set under investigation, two problem-specific descriptors have been developed. All descriptors considered in this work are represented as vectors, the individual elements of which are referred to as *features*. See the Supporting Information (Section “Descriptor properties”) for useful requirements for the development and choice of a suitable descriptor.

**The counting descriptor C**_**FG**_ reflects the number of each functional group (FG) at the *meta* positions (group 1) and the *para* positions (group 2) as well as the number of substituent combinations regarding the *meta* positions located at the same ring (group 3) and regarding the *para* positions adjacent to *meta* positions (group 4). The last two groups ensure that the descriptor is a unique description of the substitution pattern.

**Figure 4:**
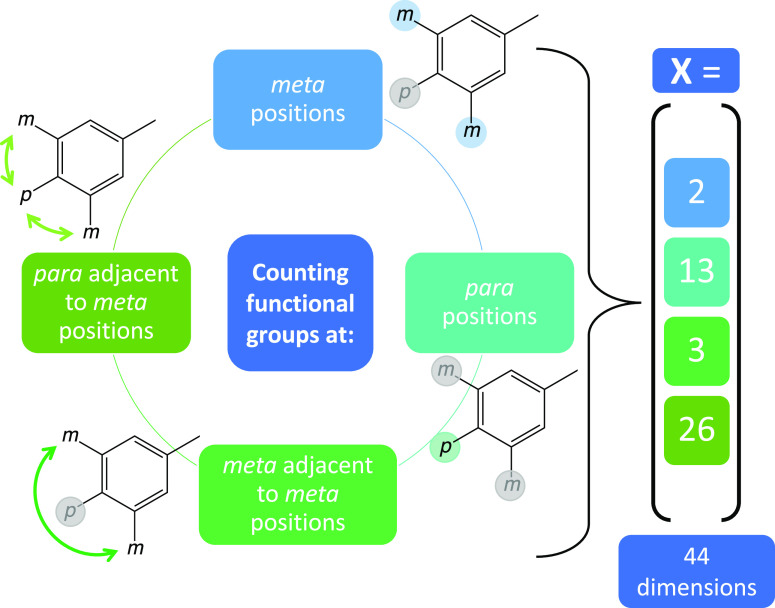
Schematic description of the counting descriptor C_FG_. The number of occurrences is counted individually for every substituent or combination of substituents according to the categories shown. The right-hand side represents the number of descriptor dimensions (44 in total) occupied by the different categories.

In Figure 4, a schematic description of C_FG_ is shown. The hydrogen atom is neglected as FG. Considering all possible substituents (*m* = 2 and *p* = 13), the descriptor dimension is composed of *m* = 2 features for group 1, *p* = 13 features for group 2, *m* × (*m* + 1)/2 = 3 features for group 3, and *m* × *p* = 26 features for group 4. This application-specific descriptor features 44 dimensions. It can be applied only to this specific data set. At the same time, it is an easy-to-interpret descriptor.

**The original *F***_**2B**_**descriptor** was proposed by Pronobis *et al.*(49) and is a general descriptor including two-body interactions. It is specified for all possible element pairs in the data set, including hydrogen atoms. For each unique element combination (*x*, *y*), the pairwise sum of inverse internuclear distances, {*R*_*ij*_}, is calculated without double counting
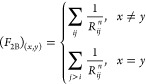
2

**Figure 5:**
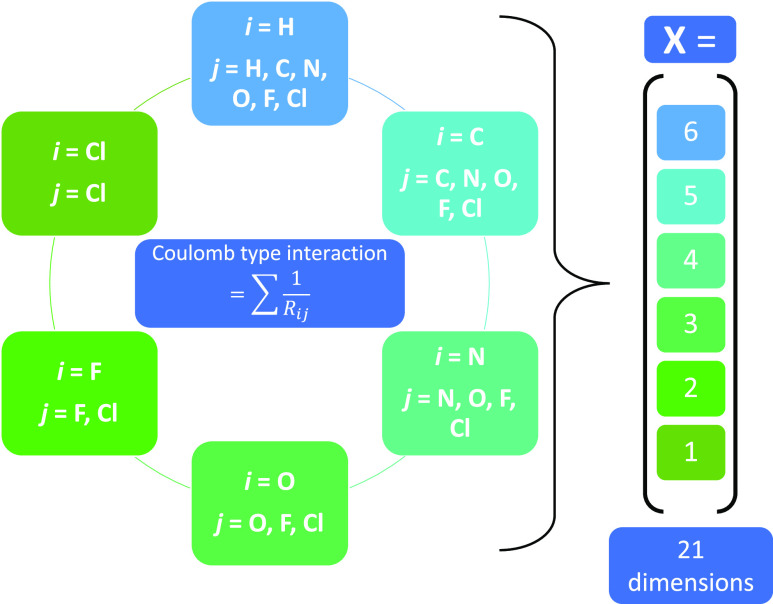
Schematic description of the *F*_2B_^1^ descriptor. The sum of pairwise Coulomb-type interactions is calculated individually for every unique element pair. The right-hand side represents the number of descriptor dimensions (21 in total) occupied by the different categories.

Therefore, the *F*_2B_ descriptor takes information on the 3-dimensional molecular structure into account; as opposed to the C_FG_ descriptor. For more flexibility, the authors of ref (49) introduced different exponents, *n* = {1, ..., 15}, resulting in 15 descriptor dimensions per unique element pair. In Figure 5, a schematic description of the *F*_2B_ descriptor is shown, which includes the Coulomb-type interactions (*n* = 1) only and is denoted as *F*_2B_^1^.

To keep the computational cost of descriptor generation as low as possible, three-dimensional molecular structures should not originate from expensive quantum chemical structure optimizations. If not otherwise mentioned, the generation of *F*_2B_-type descriptors did not include quantum chemical calculations. (The C_FG_ descriptor is independent of the actual three-dimensional structure.) See the Supporting Information Section “Structure generation” for a detailed description of the automated and quantum-chemistry-free generation of three-dimensional structures.

**Figure 6:**
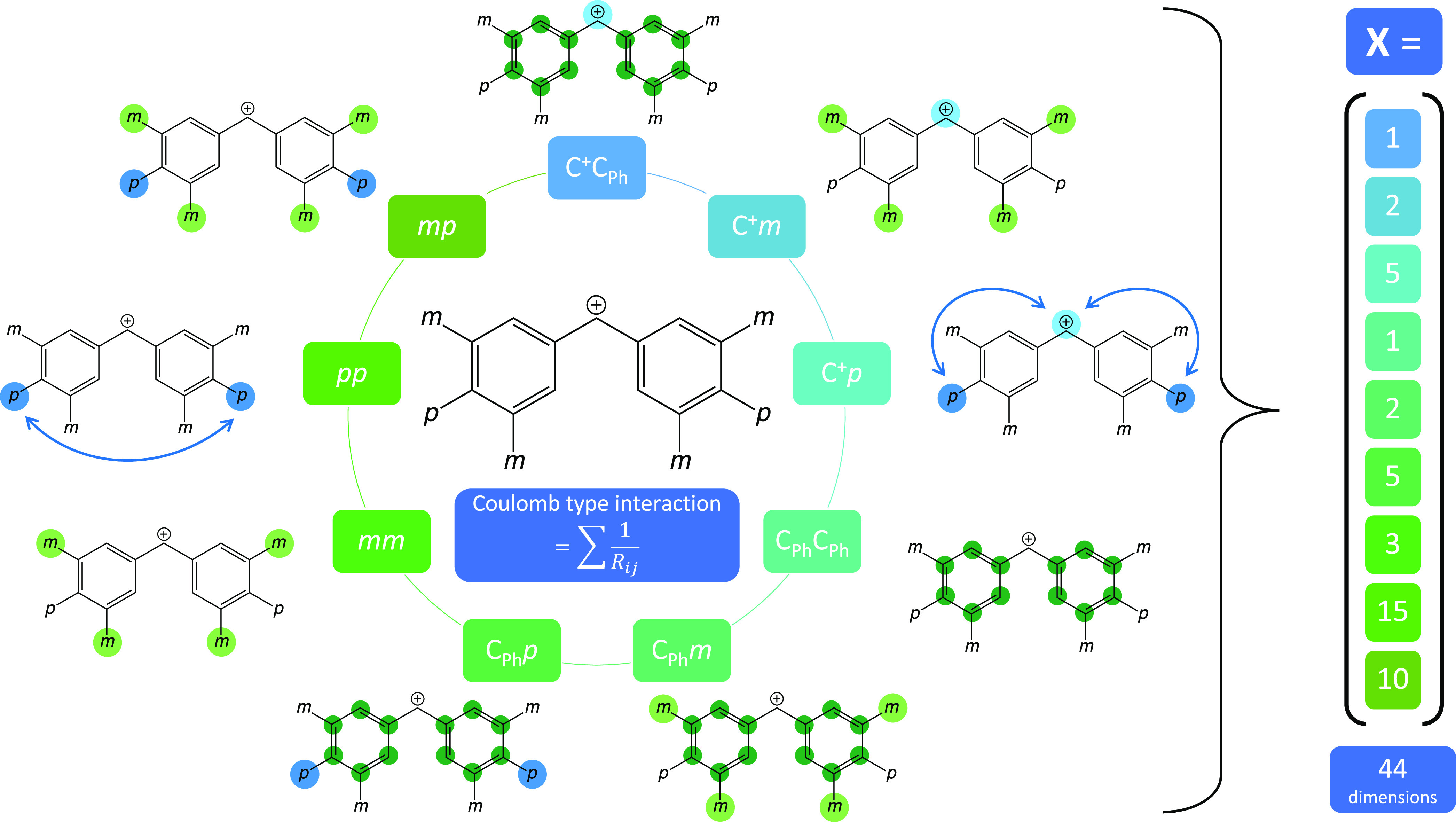
Schematic description of the *F*_2B_^split^ descriptor. In each category, the sum of pairwise Coulomb-type interactions is calculated individually for every unique element pair represented by that category. The right-hand side represents the number of descriptor dimensions (44 in total) occupied by the different categories.

**The*****F***_**2B**_^**split**^**descriptor** is an adapted version of the *F*_2B_^1^ descriptor created by us. To include more chemical information, the original *F*_2B_^1^ descriptor is divided into different interaction groups resulting from the benzhydrylium scaffold. For example, carbon atoms appear in the carbenium ion (C^+^) as well as in the phenyl rings (C_Ph_) and in different *p*-substituents (*p*-C). In *F*_2B_^1^, the interactions of these carbon atoms with a given second element are summed into a single feature. By splitting them up in the new descriptor, the interactions are divided among different regions of the molecule, which further helps in the interpretation of results. The interaction groups are shown in Figure 6. Hydrogen atoms are neglected in all of them. The descriptor dimensions sum to 44 in total. The dimensionality of this descriptor only coincidentally equals that of the C_FG_ descriptor for the underlying data set. *F*_2B_^split^ is an application-specific descriptor. It is easier to interpret than *F*_2B_^1^, but less universal.

### Quantum Molecular Properties

We selected five QMPs based on conceptual DFT^50^ as they yielded the most promising results in a data-driven investigation of electrophilicity by Hoffmann *et al.*(33) As some of them represent compositions of simpler terms, we partitioned the five QMPs to yield eight QMPs in total; see Table 2. All of them are based on energies of frontier molecular orbitals (FMOs), *i.e.*, ε_HOMO_ and ε_LUMO_, which we obtained from either quantum chemical calculations or GPR predictions.

**Table 2:** List of QMPs Examined in This Study, Including Mathematical Definitions^a^

Name		Mathematical definition
Ionization potential^51^	*	μ_FMO_^–^ = ε_HOMO_
Electron affinity^51^	1	μ_FMO_^+^ = ε_LUMO_
Global molecular hardness^52^	*	η_FMO_ = ε_LUMO_ – ε_HOMO_
Electronic chemical potential^53,54^	*	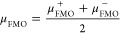
Electrophilicity index^55^	2	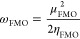
Electro-accepting power^56^	4	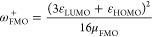
Electro-donating power^56^	5	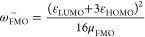
Net electrophilicity^57^	3	Δω_FMO_^±^ = ω_FMO_^+^ + ω_FMO_^–^

a The numbering of the properties corresponds to the ranking determined by Hoffmann *et al.*(33) Asterisks indicate that the corresponding QMPs are important for the definition of other QMPs of this list.

### Metrics

The metrics taken into account in this work are specified with respect to *N* observations {*y*_*i*_} and corresponding predictions . An observation *y*_*i*_ refers to either the electrophilicity parameter *E* or a QMP of the *i*^th^ molecule. The mean values of {*y*_*i*_} and  are denoted  and , respectively. The root-mean-square error (RMSE) is defined as
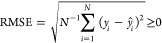
3

The coefficient of determination^58^
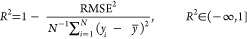
4is a strictly monotonically decreasing function of the RMSE. Both RMSE and *R*^2^ are performance metrics. Pearson’s correlation coefficient^59^
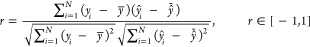
5on the other hand, is a correlation metric. While especially its squared form, *r*^2^ ∈ [0, 1], is often used as a performance metric, we emphasize that this may be a misconception. Even if two quantities correlate perfectly with each other (*r*^2^ = 1), the corresponding *R*^2^ value can be arbitrarily smaller than 1 due to a constant systematic error. Only if the least-squares solution of a linear regression problem is considered, *r*^2^ equals *R*^2^.^58^ We emphasize the importance of not confusing *R*^2^ with *r*^2^. It seems to us that there is no universal convention of whether the *R*^2^ symbol refers to a coefficient of determination or correlation, and the same holds true for the *r*^2^ symbol. We therefore recommend to always specify to which of the two quantities the symbol of choice corresponds.

### Computational Protocol

The structure-generator program was employed for the combinatorial generation of the data set structures in XYZ format with Python (version 3.9.7). After preoptimization with the xTB software (version 6.5.1)^36,60^ using GFN2-xTB,^60^ CREST (version 2.12)^61,62^ was employed to search for the most stable conformer of each molecule with the same settings as before. Full structure optimizations were then carried out with the ORCA program (version 5.0.3)^63,64^ using the hybrid meta-GGA exchange–correlation functional TPPSh^65,66^ and the D3 dispersion correction with the Becke–Johnson damping function.^67,68^ (Note that the optimized structures are not required for the generation of the structural descriptors; see the Supporting Information Section “Comparison of descriptors: guess structures versus relaxed structures”.)

The def2-SVP basis set^69^ was employed as well as the auxiliary basis set def2/J with the Coulomb integral approximation RIJCOSX.^70^ Preliminary tests motivating the choice of functional and basis set are given in the Supporting Information Section “Development of a quantum chemical protocol”.

The subsequent descriptor calculations were performed with self-written Python code, which can be accessed through the project-related GitLab repository.^71^ After preprocessing the data with Scikit-learn 0.24.2,^72^ either ordinary least-squares MLR was performed with the same package or GPR with GPy (version 1.10.0).^73^

## Results and Discussion

### Second Step (QMP to *E*)

As described in the Section “Quantum molecular properties”, eight QMPs were selected; see Table 2. Instead of selecting the single best QMP for our purposes, we propose to use a linear combination of all linearly independent terms contained in the eight preselected QMPs (six in total) to build an estimate of the electrophilicity parameter
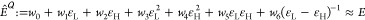
6

Here, we abbreviated ε_HOMO_ and ε_LUMO_ as ε_H_ and ε_L_, respectively. The coefficients *w*_0_ to *w*_6_ were determined by ordinary least-squares MLR and represent the intercept (*w*_0_) and the weights of the linearly independent terms (*w*_1_ to *w*_6_). For training, experimental *E* parameters of the *K* = 27 reference systems were utilized. In the following, we refer to the optimized model (eq 6) as the reference MLR (rMLR) model. Its predictions  approximate the actual electrophilicity parameter *E*, which is unknown for *M* – *K* = 3543 of the *M* = 3570 structures considered here. The relative impact of each coefficient *w*_*i*>0_ was determined by |*w*_*i*_|/∑_*j*>0_|*w*_*j*_| and the results are summarized in Table 3.

**Table 3:** Relative Impact, |*w*_*i*_|/∑_*j*_|*w*_*j*_|, of the Coefficients *w*_1_ to *w*_6_ of the rMLR Model Shown in eq 6^a^

Coefficient	Term	Value	Impact (%)	*r*
*w*_1_	ε_LUMO_	–38.1	21.1	–0.986
*w*_2_	ε_HOMO_	+22.6	12.6	–0.968
*w*_3_	ε_LUMO_^2^	–58.3	32.3	+0.980
*w*_4_	ε_HOMO_^2^	–6.8	3.8	+0.965
*w*_5_	ε_LUMO_ × ε_HOMO_	+54.3	30.1	+0.977
*w*_6_	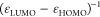	+0.2	0.1	–0.740

a The coefficients are dimensionless, as we used standardized FMO energies. Pearson’s coefficient *r* refers to the correlation between the respective term and Mayr’s *E* parameter.

The coefficients can be directly compared to each other due to standardization of FMO energies ε_FO_ (FO = HOMO, LUMO)
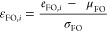
7

Here, *e*_FO,*i*_ is the raw FMO energy for the *i*^th^ molecule obtained from quantum chemical calculations, and μ_FO_ and σ_FO_ represent the mean and standard deviation of raw FMO energies, respectively, for the reference systems. As a consequence, ε_FO,*i*_ is a dimensionless quantity.

The coefficient of ε_LUMO_, *w*_1_, is quite impactful at 21.1% (third highest). The ranking by Hoffmann *et al.*,^33^ shown in Table 2, even suggests ε_LUMO_ to be the most impactful among all quantities studied by them (928 in total). The highest relative impact at 32.3% was found for the coefficient of ε_LUMO_^2^, *w*_3_, and the second highest at 30.1% was found for the product of ε_LUMO_ and ε_HOMO_, *w*_5_. Both terms were not directly considered in previous regression studies, but they are included in the electrophilicity index ω_FMO_ (see Table 2), which has been studied in related contexts^27,29−34^ and was ranked second by Hoffmann *et al.* On the other hand, the coefficients associated with the third term of the numerator (ε_HOMO_^2^) and the denominator  of ω_FMO_ are substantially less impactful, with values of 3.8% (*w*_4_) and 0.1% (*w*_6_), respectively. We draw the conclusion that the highest-impact terms of this analysis play a predominant role in correlating the electrophilicity index ω_FMO_ with the *E* parameter of benzhydrylium ions.

**Figure 7:**
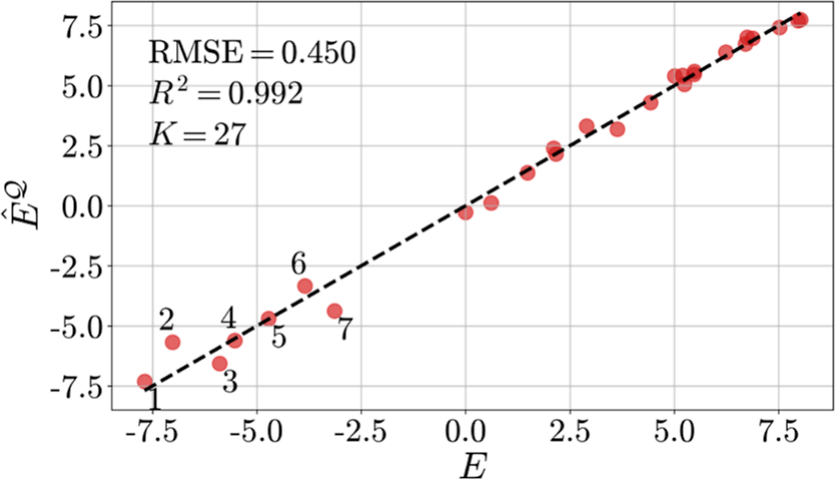
Predicted *versus* Mayr’s *E* for *K* = 27 reference structures. The coefficients of the rMLR model yielding  were optimized with respect to *E* for the same set of structures. FMO energies obtained from quantum chemical calculations served as input. See eq 6 for the mathematical definition of the rMLR model.

Figure 7 shows a plot of the rMLR-predicted  parameter *versus* its experimental analogue *E* for the reference systems. The seven structures with the lowest *E* values all comprise nitrogen-bonded *para*-substituents. They are associated with a larger deviation of  from *E* than the other structures. We assume that either increased conformational flexibility or size-related increased repulsion with *meta*-substituents (relative to the other functional groups of the data set) is responsible for this trend. Overall, the statistical test set metrics, *r*^2^ = *R*^2^ = 0.992 and RMSE = 0.450, indicate the success of the MLR approach.

The optimized rMLR model provides a reasonable starting point for implementation of the overall workflow. Additionally, given the high accuracy of the rMLR model paired with its superior interpretability, we decide against the application of more complex ML models such as neural networks,^74^ Gaussian processes,^40^ or gradient boosting decision trees.^75^ The latter was found to slightly surpass other types of ML models in the prediction of *E* parameters for a range of electrophiles including mostly carbocations and Michael acceptors.^33^

### First Step (Structure to QMP)

In the previous section (step 2), the rMLR model has been established, which connects six linearly independent QMPs with the experimentally determined electrophilicity parameter *E*. Next (step 1), we build GPR models that connect the structural descriptors C_FG_, *F*_2B_^split^, and *F*_2B_^1^ with the QMPs entering the rMLR model.

We divided the data set consisting of *M* = 3570 structures into a test set, which exclusively contains the *K* = 27 reference structures, and a training set comprising the remaining *M* – *K* = 3543 structures.

Step 1 of our workflow includes the training of two separate GPR models, one representing a structure−ε_LUMO_ relationship and the other one representing a structure−ε_HOMO_ relationship. We refer to the predictions of these models as  and , respectively. Since  is a function of only ε_LUMO_ and ε_HOMO_, substitution of the latter by their GPR-learned analogues ( and ) leads to the structure-based prediction . Note that the substitution does not alter the optimal coefficients *w*_0_ to *w*_6_ of the rMLR model. The test set performance with respect to , , and  is reported in Table 4.

**Table 4:** Test Set *R*^2^ Values (*K* = 27) for the Prediction of , , and  Obtained *via* GPR in Step 1 for All Three Descriptors^a^.

Descriptor	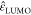	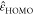	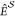
C_FG_	0.994	0.980	0.987
*F*_2B_^split^	**1.000**	**0.992**	**0.992**
*F*_2B_^1^	0.985	0.980	0.985

a The best result is shown in bold for each quantity.

**Figure 8:**
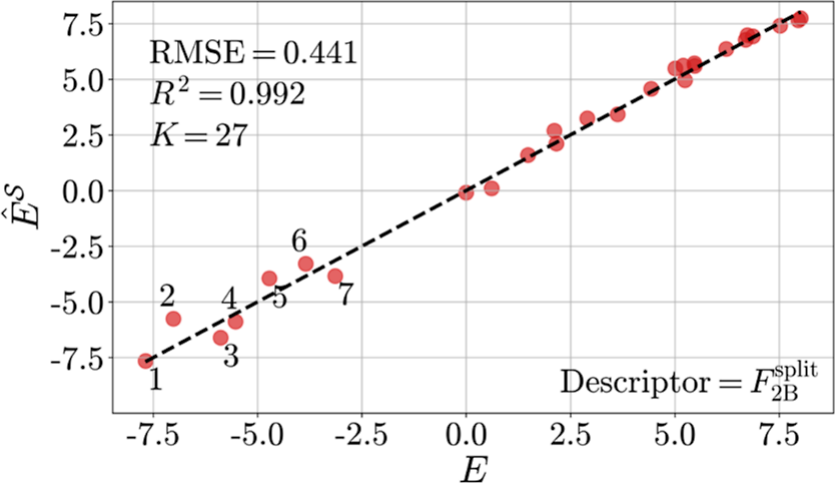
Predicted  versus Mayr’s *E* for *K* = 27 reference structures. The results are based on quantum chemical FMO energies for *M* – *K* = 3543 benzhydrylium ions.

The *F*_2B_^split^ descriptor outranks the other two descriptors in all categories, making it the descriptor of choice for prediction tasks. Consequently, *F*_2B_^split^ not only surpasses *F*_2B_^1^ in terms of interpretability but also in terms of performance. This finding also holds for C_FG_*versus F*_2B_^1^; with the exception of , where both descriptors yield the same test set accuracy. Moreover, the *F*_2B_^split^ descriptor can reproduce the LUMO energy of benzhydrylium ions exactly (up to the third decimal in *R*^2^), and the corresponding GPR predictions (Figure 8) closely resemble those of the rMLR model of step 2 (Figure 7).

**Figure 9:**
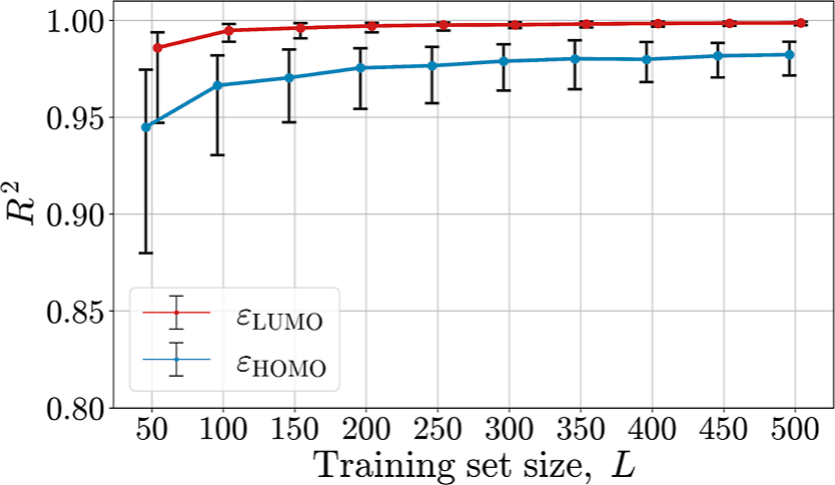
Learning curves for structure–ε_HOMO_ and structure–ε_LUMO_ relationships based on the *F*_2B_^split^ descriptor. The median of the test set *R*^2^ (*K* = 27) is shown (dots) for different training set sizes (*L* = 50, 100, ..., 500). For each size, 100 GPR models were trained on randomly selected training samples. The error bars represent 95% confidence intervals. The results suggest that the real-time reactivity prediction becomes robust and accurate (*R*^2^ consistently above 0.95) at around *L* = 200.

Finally, we would like to know if we really need *M* – *K* = 3543 systems to obtain a good prediction of *E* or whether a substantially smaller number *L* is sufficient to identify a QSRR. Learning curves are instructive for this purpose. Due to the lack of reference data, we examine learning curves for ε_HOMO_ and ε_LUMO_ obtained from quantum chemistry. Since FMO energies have been shown to yield accurate estimates of *E* (in the form of ), we consider them adequate surrogate quantities. The results for C_FG_ are shown in Figure S7. Significantly steeper learning curves were obtained for the *F*_2B_^split^ descriptor, whose performance in the prediction of electrophilicity was best compared to the other descriptors tested, see Table 4. The results for *F*_2B_^split^ (Figure 9) suggest that *L* ≈ 200 quantum chemical data points are necessary before robust and accurate predictions are obtained. In return, however, *M* – *L* ≈ 3370 or (*M* – *L*)/*M* × 100% ≈ 94% quantum-chemistry-free predictions of the electrophilicity parameter *E* can be made in real time. This result presents a proof of principle that real-time reactivity prediction is possible.

### Chemical Insights from Linear Coefficients

Interpretable models can offer a valuable understanding of patterns in data. By grasping which features of a descriptor are important for making predictions, domain experts can gain deeper insights into the problem of interest and potentially make new discoveries. Therefore, MLR models are taken into account for model interpretation in this section.

We are interested in understanding the quantitative and qualitative relationships between molecular structure (in the form of descriptors) and reactivity (*E*). Recall that we cannot use *E* directly due to a lack of data. We also cannot use  instead because it is linked with  and  but not with the structural descriptors. However,  and  are in turn linked with them. Taking into account the chemically intuitive correlation between ε_LUMO_ and *E* (see also column *r* in Table 3), we select  over  for the following analysis.

**Figure 10:**
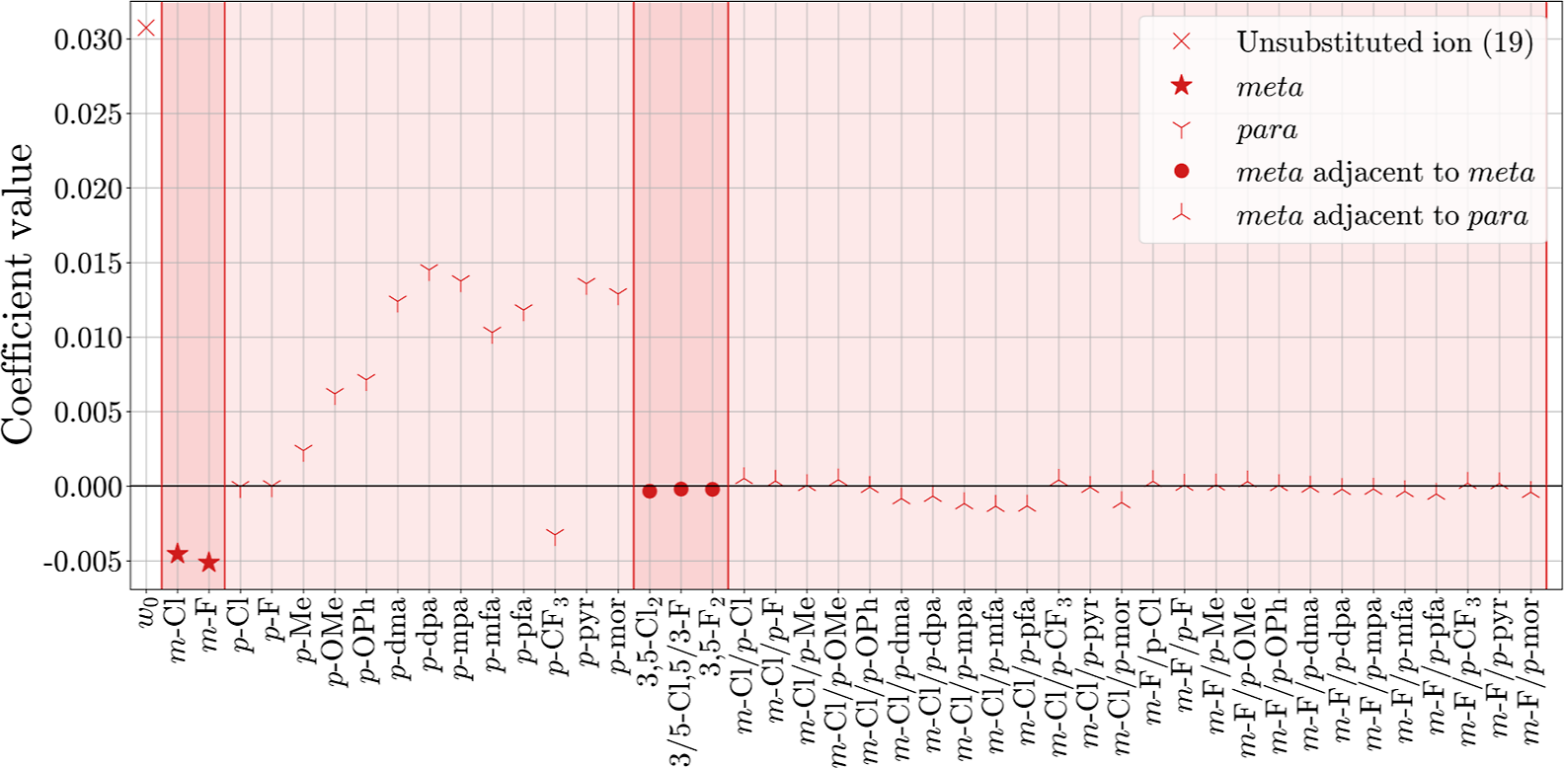
Model coefficients are shown for the regression of ε_LUMO_ on C_FG_. The different reddish sections refer to the categories in Figure 4. (Note that the two right-most sections refer to 3,5- and 3,4-substituted species, respectively.) The coefficient names are based on the notation in Mayr’s database.^21,22^

**Figure 11:**
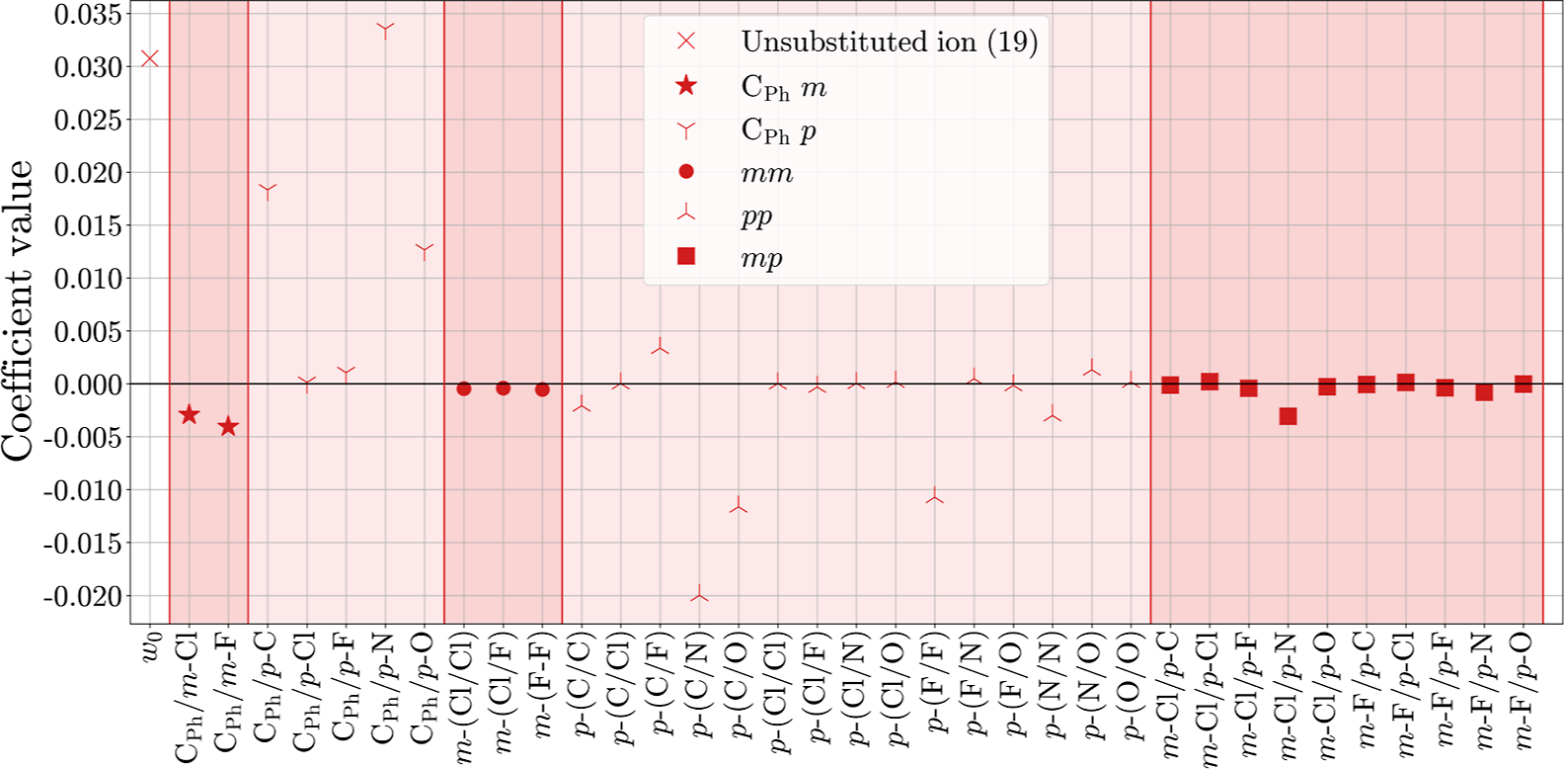
Model coefficients are shown for the regression of ε_LUMO_ on *F*_2B_^split^. The different reddish sections refer to the categories in Figure 6.

Although the GPR models perform better (cf., Tables 4 and S6), the MLR models also offer reliable predictions, allowing a reasonable analysis and interpretation of their coefficients. In Figures 10 and 11, the regression coefficients are shown for the MLR models linking C_FG_ and *F*_2B_^split^ with , respectively. To interrelate the coefficients and hence make the model more interpretable, we applied the standardization scheme of eq 7 to each feature of the two descriptors. In both cases, the intercept, *w*_0_, is an approximation to ε_LUMO_ of unsubstituted benzhydrylium ion **19** for which all other coefficients are zero.

**C**_**FG**_ (Figure 10). The different substituents at the *meta* and *para* positions of the benzhydrylium ion have the ability to push/pull electron density in/out of the aromatic rings. Negative regression coefficients correspond to electron-withdrawing groups, reducing the electron density at the carbenium ion and therefore the ε_LUMO_, as expected. This results in a larger *E* parameter (see column *r* in Table 3). For the underlying data set, large negative coefficients are primarily found for both *meta* substituents, –F and –Cl, and in the *para* position for –CF_3_. The opposite effect is found for positive regression coefficients. They correspond to electron-donating groups increasing the electron density at the carbenium ion, resulting in higher ε_LUMO_ values and smaller *E* parameters. Especially the electron-rich nitrogen- and oxygen-bonded substituents at the *para* positions substantially decrease the *E* parameter. Compared to the “*meta*” and “*para*” blocks of the C_FG_ descriptor, the coefficients of the “*meta* adjacent to *meta*” (3,5-substitution) and “*meta* adjacent to *para*” (3,4-substitution) blocks are close to zero. They are hence of minor importance for the prediction and interpretation of benzhydrylium reactivity according to the MLR results.

***F***_**2B**_^**split**^ (Figure 11). Contrary to the descriptor composition shown in Figure 6, some descriptor dimensions were deleted after performing a sensitivity analysis (see the Supporting Information Section “Sensitivity analysis of the *F*_2B_^split^ descriptor”). All coefficient blocks including the carbenium ion (C^+^) were deleted due to strong correlation with those containing the carbon atoms of the phenyl rings (C_Ph_). The decision whether to delete the C^+^ or C_Ph_ coefficient blocks is arbitrary since both possibilities lead to the same result. Additionally, the coefficient block C_Ph_/C_Ph_ was deleted as it is identical for all molecules. The first two coefficient blocks (C_Ph_/*m* and C_Ph_/*p*) describe the direct interactions of the substituents with the aromatic rings. The closer a substituent’s atom (element X) is to the phenyl rings, the greater is the effect of the X/C_Ph_ interaction on ε_LUMO_. Hence, the element that is directly bonded to the phenyl ring is expected to predominantly alter ε_LUMO_, which is consistent with the chemical intuition in many cases. The first coefficient block (C_Ph_/*m*) shows the same trend as that observed for C_FG_, with the same explanation. In the second coefficient block (C_Ph_/*p*), the interactions to *p*-Cl and *p*-N can be well interpreted since both atoms appear only in one specific position: directly bonded to the aromatic rings. For instance, the strong electron-pushing character of the nitrogen-bonded substituents is reflected by a large positive coefficient value, resulting in a high value of ε_LUMO_ and a low value of *E*. The C_Ph_/*p* interactions with carbon, fluorine, and oxygen, on the other hand, are composed of several possible positions in the molecule. Nevertheless, the general trend in the oxygen interactions can be explained: Oxygen atoms are present in three functional groups (*p*-OMe, *p*-OPh, and *p*-mor), all of which are electron-donating groups, resulting in higher ε_LUMO_ values. In the C_Ph_/*p*-F interaction block, electron-pushing effects (*e.g.*, from *p*-mfa) and electron-pulling effects (*e.g.*, from *p*-CF_3_) overlap. The number of possibilities where carbon atoms can appear in the functional groups complicates the interpretation of C_Ph_/*p*-C even more than in the previous C_Ph_/*para* interactions. In the *p*/*p* and *m*/*p* coefficient blocks, many different effects overlap, which does not allow for straightforward interpretation. Contrary to the analogous C_FG_ coefficients, some of the *p*/*p* and *m*/*p* coefficients of *F*_2B_^split^ exhibit high values, showing the limited interpretability of the latter descriptor.

In summary, the interpretation of C_FG_ is straightforward for each substituent. *F*_2B_^split^, on the other hand, can reveal details beyond simple substituent identities. At the same time, many other details of *F*_2B_^split^ are not accessible. In turn, the more universal *F*_2B_^1^ descriptor is not nearly as simple to interpret as *F*_2B_^split^. For instance, no distinction between *meta* and *para* halogen atoms is possible. In general, the more complex the descriptor structure, *i.e.*, the more different effects overlap in one descriptor dimension, the more difficult the chemical interpretation becomes. This is especially true for the “*meta* adjacent to *meta*/*para*” blocks of C_FG_ and the *p*/*p* and *m*/*p* blocks of *F*_2B_^split^.

### Exploring the Limits of Chemical Intuition

**Figure 12:**
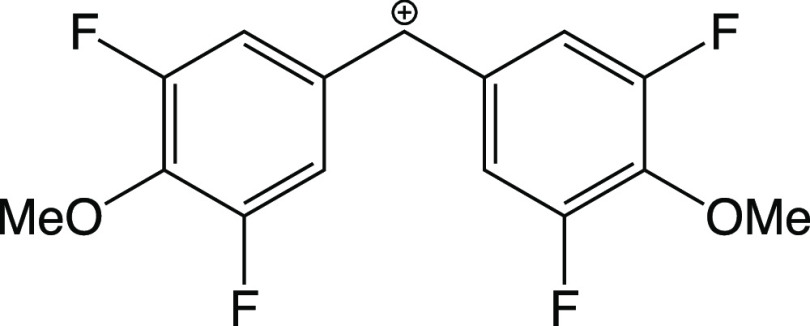
Example of a fully *m*, *p*-substituted benzhydrylium ion. Does the electrophilicity increase or decrease with respect to the unsubstituted ion **19**?

Finally, we highlight one of the practical benefits of our approach. Figure 12 shows a fully *m*, *p*-substituted benzhydrylium ion. It comprises four electron-withdrawing groups (*m*-F) and two electron-donating groups (*p*-OMe). Does the electrophilicity increase or decrease with respect to unsubstituted ion **19**? Hammett σ_*m*_ and σ_*p*_^+^ parameters suggest that the *para*-methoxy group is slightly more than twice as electron-donating (σ_*p*_^+^ = −0.78) as the *meta*-fluorine atom is electron-withdrawing (σ_*m*_ = 0.34).^48^ Assuming additivity, these values suggest that, overall, the electrophilicity of the fully *m*, *p*-substituted ion should slightly decrease: 4·σ_*m*_(F) + 2·σ_*p*_^+^(OMe) = −0.20. With the quantitative approach presented in this study, applying GPR in combination with *F*_2B_^split^, we observed the following trend.

**Figure 13:**
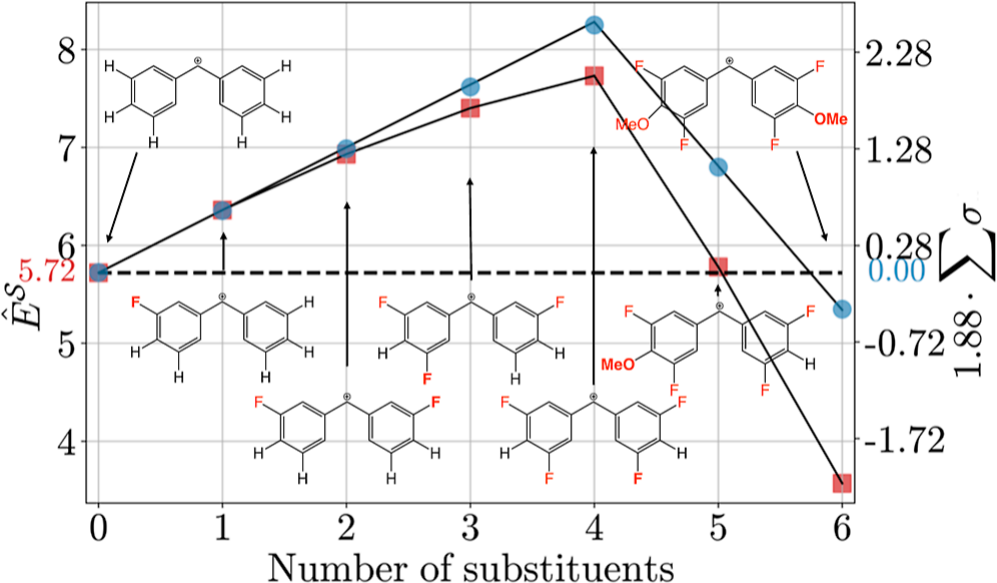
Change in  (red squares) and a scaled sum of Hammett σ parameter (blue dots) with an increasing level of substitution for the benzhydrylium ion shown in Figure 12. The sum of the Hammett parameters is multiplied by a scaling factor of 1.88 (obtained from the first two data points chosen as reference) to allow comparison between red and blue values. The dashed horizontal line indicates values of  (5.72, red) and 1.88 × ∑σ (0.00, blue) for the unsubstituted benzhydrylium ion **19**, respectively. In the displayed structures, added functional groups are highlighted in red, with the most recent group in bold.

Without the electron-donating *para* groups, the fully *meta*-fluorinated benzhydrylium ion is more electrophilic (*E* = 7.73) than the unsubstituted prototype **19** (*E* = 5.72). Adding both *p*-OMe group, its electrophilicity decreases by −4.16 units (*E* = 3.57) and hence below the prototype’s value. In Figure 13, the quantitative change in  (red squares) and the scaled sum of Hammett σ parameters (blue dots) for each individual substituent addition is shown. As indicated by the approximately linear trends in , the electrophilicity increases/decreases by a substituent-specific, Hammett-like value when the same substituent is added in the same position type. However, compared to the scaled sum of Hammett σ parameters, the latter shows an actual linear relationship that overestimates the  parameters. The deviation is moderate for the *meta* additions, but larger for the *para* additions. Assuming additivity of Hammett σ parameters, the decrease in *E* of the fully *m*,*p*-substituted ion is expected to be small compared to that of **19**, whereas this work predicts a larger effect. A possible conclusion is that the validity of the additivity assumption for the Hammett σ parameters decreases with an increasing degree of substitution. That the differences in *E* decrease with higher substitution is also evident from the experimental values listed in Table 1, see the series **19**, **21**, **23**/**24**, **25**, **27**. Therefore, the prediction framework presented in this work has an advantage over Hammett σ parameters in that it takes into account the experimentally observed trends for highly substituted rings.

## Conclusions and Outlook

We have explored the feasibility of real-time, data-driven reactivity prediction for routine synthesis planning. In previous data-driven reactivity studies, QMPs were used to learn quantitative relationships between these properties and Mayr-type reactivity parameters (*E*, *N*, *s*_N_). While QMPs are informative quantities, their calculation usually is computationally intensive, preventing the possibility of a real-time approach.

As an alternative, we have considered structural descriptors that can be generated in real time. A combinatorial data set of *M* = 3570 benzhydrylium ions served as the domain of application. For only *K* = 27 of these systems, electrophilicity parameters *E* are available. For each system, three structural descriptors were generated, ranging from application-specific but interpretable (C_FG_, *F*_2B_^split^) to application-agnostic but less interpretable (*F*_2B_^1^). However, a direct mapping of the structural descriptors to *E via* some regression method is not possible due to lack of data [see the Supporting Information Section “The direct path (structure to *E*)”].

Instead, we developed a two-step workflow based on the GPR (step 1) and MLR (step 2) techniques. Step 2 of the workflow resembles previous approaches: a quantitative QMP–*E* relationship is learned based on *K* training data points. The QMPs considered here are functions of FMO energies. In step 1 of the workflow, quantitative descriptor–QMP relationships are learned to replace the expensive QMP generation by efficient real-time predictions. We identified *F*_2B_^split^ to be the descriptor of choice with respect to the rate of learning. Our analysis suggests that *L* ≈ 200 training data points (*i.e.*, quantum chemical calculations) are necessary to make robust and accurate *E* predictions with a test set *R*^2^-value of approximately 0.99. Hence, we can replace quantum chemical calculations with real-time predictions for almost 94% of all benzhydrylium ion structures. In summary, the two-step workflow is an effective approach if *K* ≪ *L* ≪ *M*.

The comparison of our predictions of *E* with the sum of Hammett σ parameters reveals that the validity of the additivity assumption of the latter decreases with an increasing degree of substitution.

The next challenge on the way to real-time reactivity prediction for arbitrary molecules is to extend our approach to a broader range of structural classes. However, even within the benzhydrylium space, many more substituents are to be explored. We assume that the second step of our approach is—without any further modification—applicable to other functional groups and the resulting substitution patterns.

At the same time, we need to overcome the problem of data shortage. With the information provided by data-driven reactivity studies, synthetic chemists can more systematically plan new experiments that in turn feed future data-driven campaigns. We invite laboratories around the globe to help us build such experimental–computational feedback loops to accelerate advances in organic synthesis.

## Supporting Information Available

Further discussion on structure generation, descriptors, and results and a Git repository to compile the python code, the data set, and the model data (PDF)
