# Supplementary material for: Quantitative Structure–Reactivity Relationships for Synthesis Planning: The Benzhydrylium Case
Source: J Phys Chem A. 2023 Dec 19;128(1):343–54. doi: 10.1021/acs.jpca.3c07289 (PMC10788916; doi:10.1021/acs.jpca.3c07289)
Supplement: Supplementary file 1 — jp3c07289_si_001.pdf [file jp3c07289_si_001.pdf]

Supporting Information - Quantitative Structure–Reactivity Relationships for Synthesis Planning: The Benzhydrylium Case

Maike Eckhoff,† Johannes V. Diedrich,†,‡ Maike Mücke,†,‡ and Jonny Proppe*,†

†*Institute of Physical and Theoretical Chemistry, TU Braunschweig, 38106 Braunschweig, Germany*

‡*Institute of Physical Chemistry, University of Göttingen, 37077 Göttingen, Germany*

E-mail: j.proppe@tu-braunschweig.de

More supporting information can be accessed through the project-related GitLab repository: `https://git.rz.tu-bs.de/proppe-group/qsrr-benzhydrylium`.

Structure generation

The generation of the combinatorial data set of benzhydrylium ion derivatives has been carried out with the `structure-generator` program, a self-written Python code, which can be accessed through the project-related GitLab repository.[1] The generated structures are named according to the scheme $meta_{11}\_para_1\_meta_{12}\_\_meta_{21}\_para_2\_meta_{22}$. The first index specifies the ring. The second index differentiates between the two possible *meta* positions for each ring.

Three files are needed to run the program. The first file, `basic_framework.xyz`, represents the unsubstituted benzhydrylium ion, H_H_H__H_H_H. It is important that the right order-

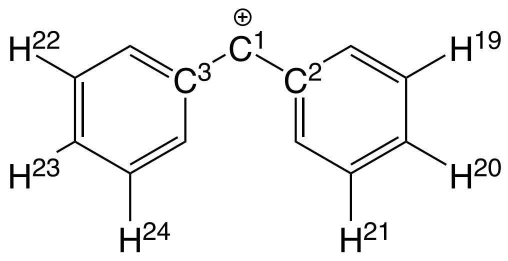

Figure S1: Atom indices required as input for the structure generation process. The numbers reflect the ordering of lines in the file `basic_framework.xyz`.

ing of atoms is considered as the program relies on ordered information. In Fig. S1, the correct ordering is shown. The first three atoms are the carbon atoms connecting the phenyl rings to the carbenium ion. The last six atoms (19–24) belong to the *meta* and *para* hydrogen atoms. To ensure efficient structure generation, the molecular scaffold was fixed for the following steps. The *meta* substituents were placed at the same position replacing the *meta* hydrogen atoms. Each of the 13 possible substituents (Section "Data set" of the main text) was placed in pairs at the *para* positions. After restrained optimization of the substituents, the coordinates were written to two separate files, `para_1_functional_groups.txt` and `para_2_functional_groups.txt`, respectively, each representing one of the aromatic rings. In the program itself, the names of the possible *p*-substituents as well as the number of atoms of each substituents need to be specified in lists. Due to the fixed scaffold, arbitrary combinations of the *p*-substituents can be realized without having to expect structural distortions.

All possible substitution combinations including four *meta* and two *para* positions were generated. $3^4 \cdot 14^2 = 15876$ structures were obtained this way. Duplicates generated by the symmetry axes were sorted out, leaving a final set of 3570 unique benzhydrylium ion derivatives.

Examination of rotational symmetry

As shown in Fig. 3 (main text) with dashed gray lines, we assummed approximate C_2 symmetry for the rotation axes passing through the single bonds connecting the carbenium ion to the aromatic rings of the benzhydrylium structures. To show that this assumption is reasonable, we took two example molecules, F_H_H__H_H_H and H_H_F__H_H_H, which are supposed to be identical within this approach. For both molecules, $\varepsilon_{\mathrm{LUMO}}$ and $\varepsilon_{\mathrm{HOMO}}$ were calculated and compared to the respective total range of values within the data set, see Figs. S2 and S3.

The assumption of rotational symmetry appears reasonable, because the values for $\varepsilon_{\mathrm{LUMO}}$

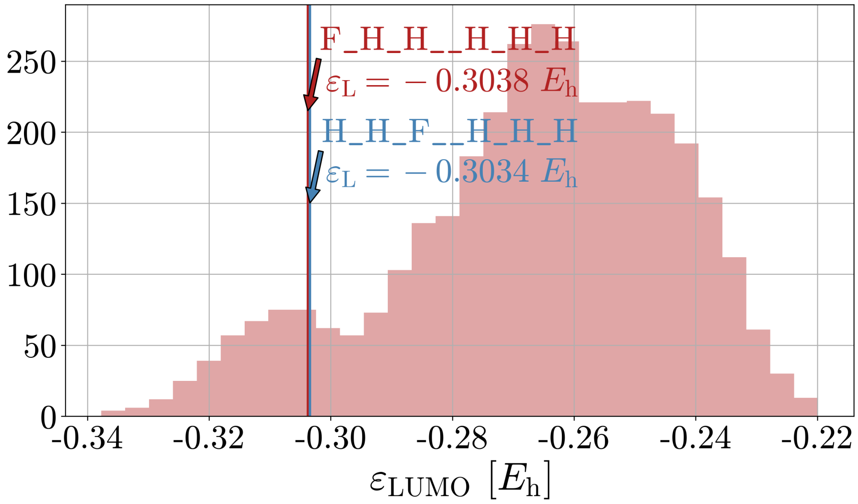

Figure S2: Histogram of $\varepsilon_{\mathrm{LUMO}}$ values contained in the curated benzhydrylium data set. In red, $\varepsilon_{\mathrm{LUMO}}$ is shown for the molecule F_H_H__H_H_H, and in blue, $\varepsilon_{\mathrm{LUMO}}$ is shown for the molecule H_H_F__H_H_H.

and $\varepsilon_{\mathrm{HOMO}}$ are only negligibly different compared to the total range covered within the data set. Furthermore, since all *meta*-substituents represent single-atom functional groups that do not induce sterically complex environments, we expect this assumption to be applicable to all structures of the data set.

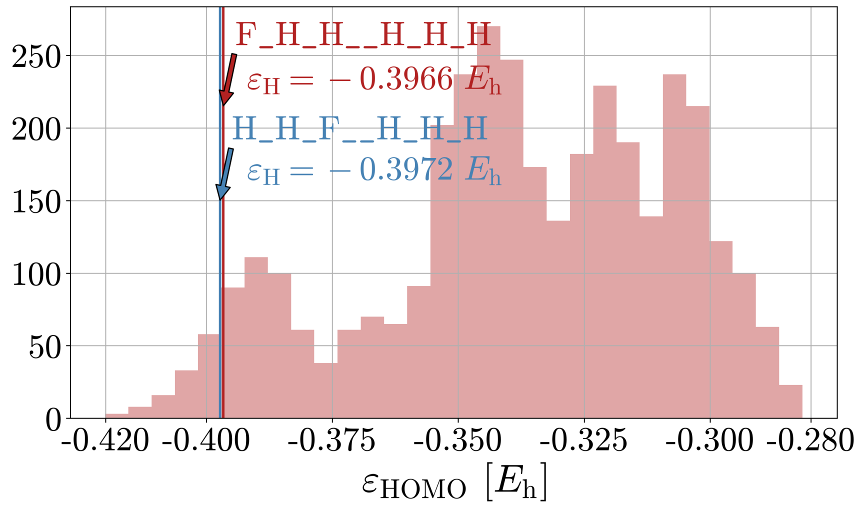

Figure S3: Histogram of ε_{HOMO} values contained in the curated benzhydrylium data set. In red, ε_{HOMO} is shown for the molecule F_H_H__H_H_H, and in blue, ε_{HOMO} is shown for the molecule H_H_F__H_H_H.

Development of a quantum chemical protocol

Structural analysis

To ensure that the reference quantum chemical calculations of the benzhydrylium ion derivatives are as accurate as necessary, a specific, well-tested computational protocol needs to be developed. For this purpose, the available crystal structures of five amino-substituted benzhydrylium ion derivatives were considered:[2] For $(dma)_2CH^+$ (**2**), $(ind)_2CH^+$, $(jul)_2CH^+$, $(lil)_2CH^+$, and $(pyr)_2CH^+$ (**1**). For these structures, different molecular and computational settings were varied: calculations with/without counterion, calculations with/without acetonitrile solvent molecules (implicit and hybrid treatment), and gas phase calculations. Additionally, different exchange–correlation (XC) functionals and further calculation settings were tested.

All calculations were performed using the ORCA program (version 4.2.1).[3,4] The computational settings tested include the XC functionals TPSS and TPSSh[5,6] employing the def2-TZVPP basis set[7] and the D3 dispersion correction with the Becke–Johnson damping function.[8,9] For the analysis of the different quantum chemical protocols, relevant geometric

parameters were extracted for comparison to experimental data by Mayer et al.:[2] the carbon

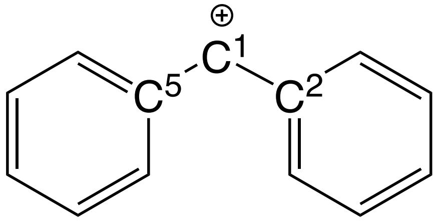

Figure S4: Carbon atom labeling for the benzhydrylium framework based on the work of Mayer et al.[2]

bond lengths C^1–C^2 and C^1–C^5, the averaged bond length, and the bond angle C^2–C^1–C^5. In Fig. S4, the labeling of the atoms is shown as numbered in ref.[2] The calculated averaged bond length is very similar to both the X-ray data and the computational data of Mayer et al. for both XC functionals tested, especially for the D3BJ-TPSSh/def2-TZVPP calculations. However, the X-ray determined bond angles could not be reproduced by any of the tested protocols.

The influence of the angle differences was then investigated with a sensitivity analysis. For this, two different structure types were taken for the five benzhydrylium structures: First, the relaxed optimized structure with a constrained C^2–C^1-C^5 angle value which is taken from the crystal structure, and second, the fully relaxed structure. For both structures, the values of the five global quantum molecular properties (QMPs) (see Section "Quantum mechanical properties" of the main text) were calculated and compared for each molecule. With this setting, it can be approximated that the differences in energy come (mainly) from the angle, because the angle is the predominant difference between both structure types. Linear regression of E[10] on each QMP for the $K = 27$ reference structures and 4 additional benzhydrylium ions of Mayr's database[11,12] was performed. Two different RMSE values were calculated, see Table S1. First, RMSE values based on the results for fully optimized structures compared to results for crystal structure angles. Second, the overall RMSE of the regression of E on the different QMPs. The resulting energy difference for different angles (first RMSE type) is smaller than the error obtained from the calculations (second RMSE

type), and therefore negligible.

Table S1: RMSE values for the regression of E on QMPs for two different structure types: DFT optimized structures (opt) and DFT optimized structures with a fixed C^2–C^1–C^5 angle taken from crystal structures (cryst). For mathematical definitions of QMPs, see Table 2 of the main text.

QMP	μ^+_{FMO}	ω_{FMO}	$\Delta\omega^{\pm}_{FMO}$	ω^+_{FMO}	ω^-_{FMO}
RMSE(opt, cryst)	0.207	0.380	0.394	0.363	0.379
$RMSE_{overall}$	0.980	2.71	2.95	2.48	2.70

Basis set analysis

The smaller basis set def2-SVP[7] compared to def2-TZVPP was examined by comparing results of the regression of E on each QMP, which are summarized in Table S2. The results (printed in bold) for QMP 1 = ε_{LUMO} (ranked highest global QMP in the study by Hoffmann et al.[13]) only slightly differ between both basis sets. In summary, the gain in computing time by using def2-SVP as basis set compared to the slightly higher accuracy by using def2-TZVPP has led us to the decision of choosing def2-SVP as basis set for the final quantum chemical protocol. The developed quantum chemical protocol comprises D3BJ-TPSSh/def2-SVP def2-RIJCOSX in Orca.

Table S2: R^2 values for the regression of E parameter[10] on different QMPs, calculated with TPSSh-D3BJ, for $K + 4 = 31$ structures.

QMP	μ^+_{FMO}	ω_{FMO}	$\Delta\omega^{\pm}_{FMO}$	ω^+_{FMO}	ω^-_{FMO}
def2-SVP	**0.9756**	0.8672	0.8337	0.8337	0.8693
def2-TZVPP	**0.9757**	0.8872	0.8575	0.9113	0.8890

These preliminary tests were performed with the version 4.2.1 of ORCA.[3,4] Subsequent calculations were performed with version 5.0.3 of Orca.[3,14] We assume that the test results can be transferred to the new version of the program.

Consistency between TPPSh and B3LYP

The structure optimization protocol developed for the benzhydrylium data set was examined for consistency in $\varepsilon_{\mathrm{LUMO}}$ and $\varepsilon_{\mathrm{HOMO}}$ for three different DFT settings. For this purpose, two XC functionals, the hybrid meta GGA TPSSh from the developed protocol and the hybrid functional B3LYP,[15,16] were selected. For each functional the structure optimizations were performed for the $K + 4 = 31$ benzhydrylium structures mentioned above. Additionally, B3LYP single point calculations were performed on the TPSSh-optimized structures. The resulting $\varepsilon_{\mathrm{LUMO}}$ and $\varepsilon_{\mathrm{HOMO}}$ of the optimizations showed very little variation between the different cases. For better analysis, regression of E was performed on $\varepsilon_{\mathrm{LUMO}}$ and $\varepsilon_{\mathrm{HOMO}}$, respectively. The corresponding R^2 values are summarized in Table S3. These results demonstrate the consistency of two different XC-functional types chosen for the calculation of $\varepsilon_{\mathrm{LUMO}}$ and $\varepsilon_{\mathrm{HOMO}}$.

Table S3: R^2 values of the regression of E on $\varepsilon_{\mathrm{LUMO}}$ and $\varepsilon_{\mathrm{HOMO}}$, respectively. Structure optimizations for $K+4 = 31$ benzhydrylium structures were performed with TPSSh-D3BJ/def2-SVP, B3LYP-D3BJ/def2-SVP//TPSSh-D3BJ/def2-SVP, and B3LYP-D3BJ/def2-SVP.

	$\varepsilon_{\mathrm{LUMO}}$	$\varepsilon_{\mathrm{HOMO}}$
TPSSh-D3BJ/def2-SVP	0.975	0.947
B3LYP-D3BJ/def2-SVP//TPSSh-D3BJ/def2-SVP	0.975	0.949
B3LYP-D3BJ/def2-SVP	0.973	0.945

Descriptor properties

Finding a proper structural descriptor for a given problem can be a difficult task. The following requirements are useful for developing or choosing suitable descriptors:[17] The descriptor should be invariant towards translational **(a)** and rotational **(b)** transformations of a molecule. Additionally, permutations of atomic indices should not change any properties of the molecule: the descriptor needs to be permutationally invariant **(c)**. Many ML models also require descriptors to be invariant with respect to the number of atoms contained in a molecule **(d)**. Continuity and differentiability **(e)** are mathematical requirements, which help

to simplify the optimization in feature space. Furthermore, small changes in the structure should lead to small changes in the descriptor, and similar molecules should be described by similar descriptors. A compact **(f)** descriptor should contain as much information as needed while keeping the descriptor dimensions as low as possible. The computational effort to construct the descriptor should be significantly lower than producing the predictions by other methods, e.g., quantum chemical calculations **(g)**. Finally, a unique structure-to-descriptor mapping of a molecule **(h)** is desired. The more requirements are fulfilled, the better the descriptor in terms of prediction efficiency and accuracy. Fulfilling all conditions has not yet been achieved, requiring consideration of which is most relevant to the specific problem. See Table S4 for a comparison of the descriptors under investigation with respect to these requirements.

Table S4: Characterization of the descriptors under investigation with respect to the requirements specified in the text.

	C_{FG}	F_{2B}^{1}	F_{2B}^{split}
(**a**)	✓	✓	✓
(**b**)	✓	✓	✓
(**c**)	✓	✓	✓
(**d**)	✓	✓	✓
(**e**)	x	✓	✓
(**f**)	✓	✓	x
(**g**)	✓	✓	✓
(**h**)	x	x	x

Comparison of descriptors: guess structures versus relaxed structures

To keep the computational cost as low as possible, the three-dimensional structures should not originate from expensive quantum chemical structure optimizations. Therefore, non-optimized XYZ coordinates out of the `structure-generator` program were taken for the descriptor generation of F_{2B}^{split} and F_{2B}^{1} in this work. To compare the quality of the results for

optimized (relaxed) and non-optimized (guess) XYZ coordinates, F_{2B}^{split} serves as an example. The results are summarized in Table S5. Both structure types yield very similar results.

Table S5: R^2 values for the prediction of $\varepsilon_{\mathrm{LUMO}}$, $\varepsilon_{\mathrm{HOMO}}$, and $\hat{E}^{\mathcal{S}}$ of $K = 27$ reference structures with F_{2B}^{split} based on non-optimized and optimized XYZ coordinates.

Descriptor based on	$\varepsilon_{\mathrm{LUMO}}$	$\varepsilon_{\mathrm{HOMO}}$	$\hat{E}^{\mathcal{S}}$
guess structures	1.000	0.992	0.992
relaxed structures	0.999	0.991	0.992

Based on these tests, we can confirm that the choice of inexpensive guess structures over CPU-intensive relaxed structures is reasonable.

Sensitivity analysis of the F_{2B}^{split} descriptor

The importance of the different C^+ and C_{Ph} interaction groups (IGs) of the F_{2B}^{split} descriptor is investigated. Due to the structure generation process, the distances between atoms in the $C_{\mathrm{Ph}}/C_{\mathrm{Ph}}$ and C^+/C_{Ph} IGs are identical in all structures. Therefore, these IGs are not important for the description of the data set and were deleted. Fig. S5 shows the correlation matrix for the first 16 dimensions of F_{2B}^{split} (C_{Ph}/X and C^+/X IGs) skipping the intercept w_0. A strong correlation between the interactions of C^+/X to C_{Ph}/X and vice versa can be seen. For the application of the F_{2B}^{split} descriptor, all C^+/X interactions were deleted.

The direct path (structure to $\boldsymbol{E}$)

To verify the necessity of our two-step approach, we now examine whether structure–E relationships can be learned directly, i.e., without detouring through quantum chemical calculations. We used the descriptors considered in this work to train GPR models on all available E parameters, which is a small number ($K = 27$). The results are shown in Fig. S6.

The test set comprises the remaining $M - K = 3543$ structures of the data set. Due to the unavailability of experimental data for these structures, it was assumed that the rMLR model predictions $\hat{E}^{\mathcal{Q}}$ would provide adequate surrogate values for E. The test set

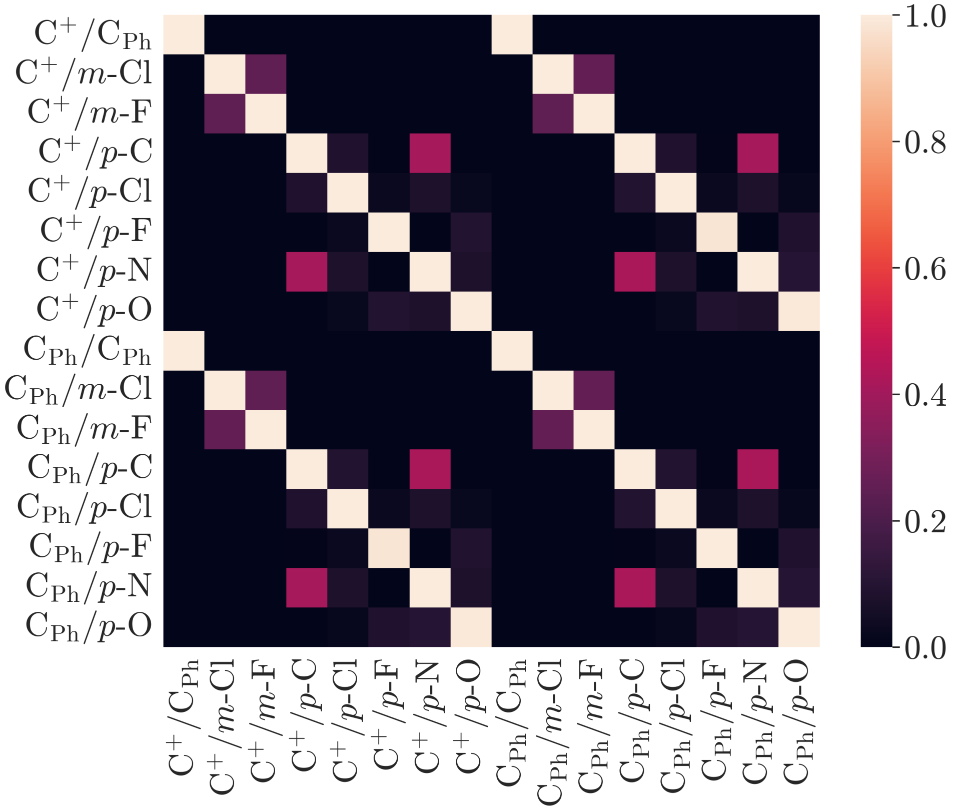

Figure S5: Correlation matrix for the F_{2B}^{split} descriptor dimensions including C^+/X and C_{Ph}/X interactions.

metrics, $R^2 = -0.276$ and RMSE $= 3.208$, confirm that it is not sufficient to perform reliable predictions based on a training set including only 27 systems.

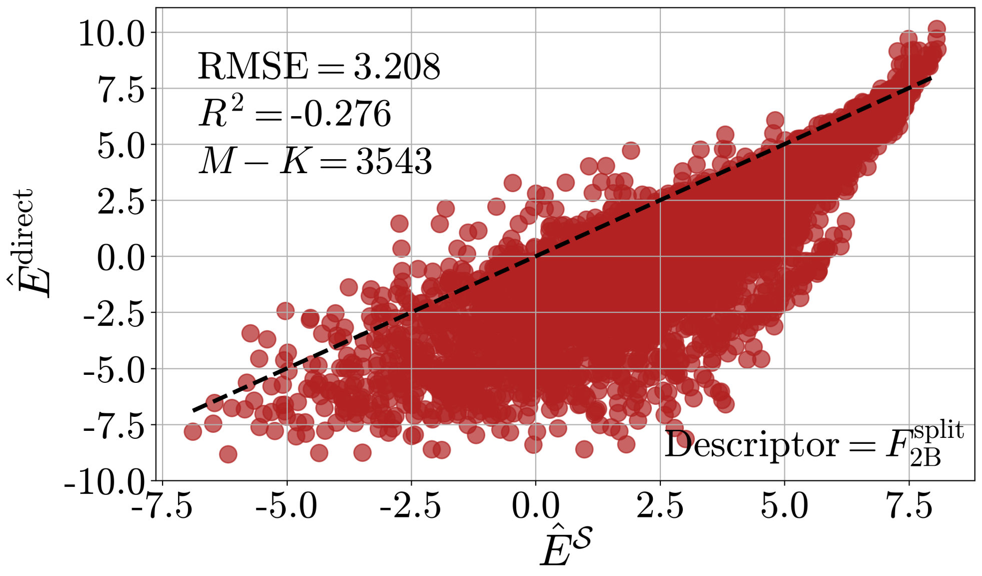

Figure S6: $\hat{E}^{\mathrm{direct}}$ versus $\hat{E}^{\mathcal{S}}$ for $M - K = 3543$ benzhydrylium ions. The results are based on a direct mapping of the $F_{2\mathrm{B}}^{\mathrm{split}}$ descriptor onto Mayr's E for $K = 27$ reference structures.

Miscalleneous tables and figures

Table S6: Test set R^2 values ($K = 27$) for the prediction of $\hat{\varepsilon}_{\text{LUMO}}$, $\hat{\varepsilon}_{\text{HOMO}}$, and $\hat{E}^{\mathcal{S}}$ obtained via MLR in step 1 for all three descriptors. The best result is shown in bold for each quantity.

Descriptor	$\hat{\varepsilon}_{\text{LUMO}}$	$\hat{\varepsilon}_{\text{HOMO}}$	$\hat{E}^{\mathcal{S}}$
C_{FG}	0.982	0.933	0.966
$F_{\text{2B}}^{\text{split}}$	**0.992**	**0.959**	**0.975**
F_{2B}^{1}	0.979	0.955	0.970

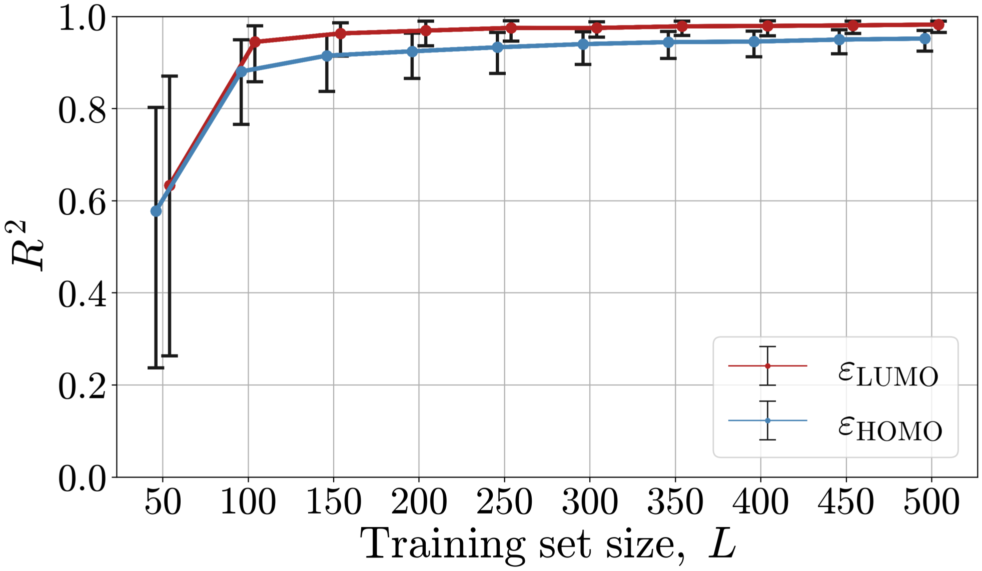

Figure S7: Learning curves for structure-$\varepsilon_{\text{HOMO}}$ and structure–$\varepsilon_{\text{LUMO}}$ relationships based on the C_{FG} descriptor. The median of the test set R^2 ($K = 27$) is shown (dots) for different training set sizes ($L = 50, 100, 150, ..., 500$). For each size, 100 GPR models were trained on randomly selected training samples. The error bars represent 95% confidence intervals. The results suggest that real-time reactivity prediction becomes robust and accurate at around L =350.

Table S7: Translation table. IDs of benzhydrylium ions used in this work are translated to IDs used in previous work.[18] The electrophilicity parameter E^{19} for each structure is shown.

ID, here	ID, Ref.[18]	E^{19}	ID, here	ID, Ref.[18]	E^{19}
**1**	**E5**	−7.69	**15**	**E21**	4.43
**2**	**E6**	−7.02	**16**	**E22**	5.01
**3**	**E7**	−5.89	**17**	**E23**	5.20
**4**	**E8**	−5.53	**18**	**E24**	5.24
**5**	**E9**	−4.72	**19**	**E25**	5.47
**6**	**E10**	−3.85	**20**	**E26**	5.48
**7**	**E11**	−3.14	**21**	**E27**	6.23
**8**	**E15**	0.00	**22**	**E28**	6.70
**9**	**E16**	0.61	**23**	**E29**	6.74
**10**	**E17**	1.48	**24**	**E30**	6.87
**11**	**E18**	2.11	**25**	**E31**	7.52
**12**		2.16	**26**	**E32**	7.96
**13**	**E19**	2.90	**27**	**E33**	8.02
**14**	**E20**	3.63			

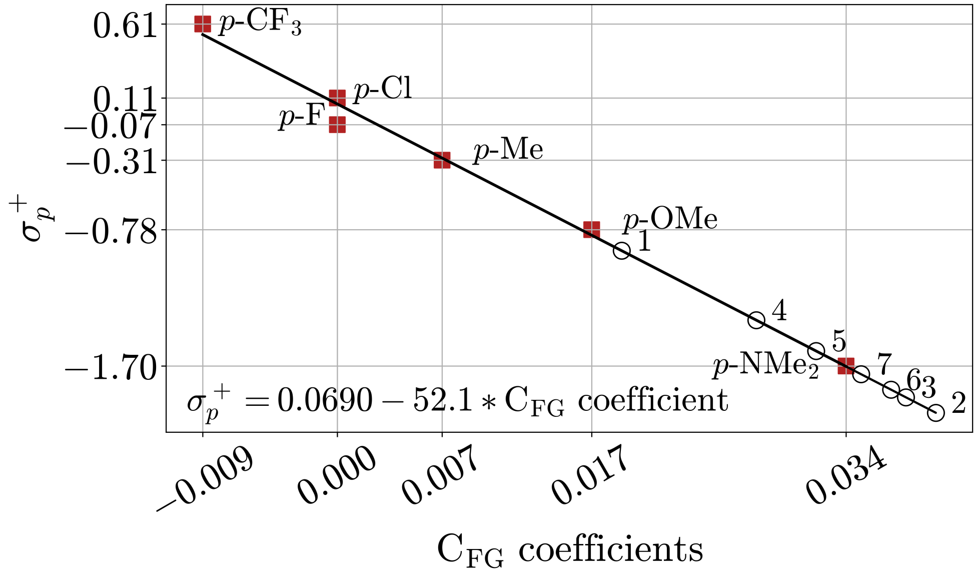

Figure S8: Correlation of Hammett–Brown σ_p^+ parameters with the linear coefficients of the C_{FG}–ε_{LUMO} MLR model. The blank dots represent newly predicted σ_p^+ parameters for *para*-substituents present in the data set. IDs are assigned in Table S8.

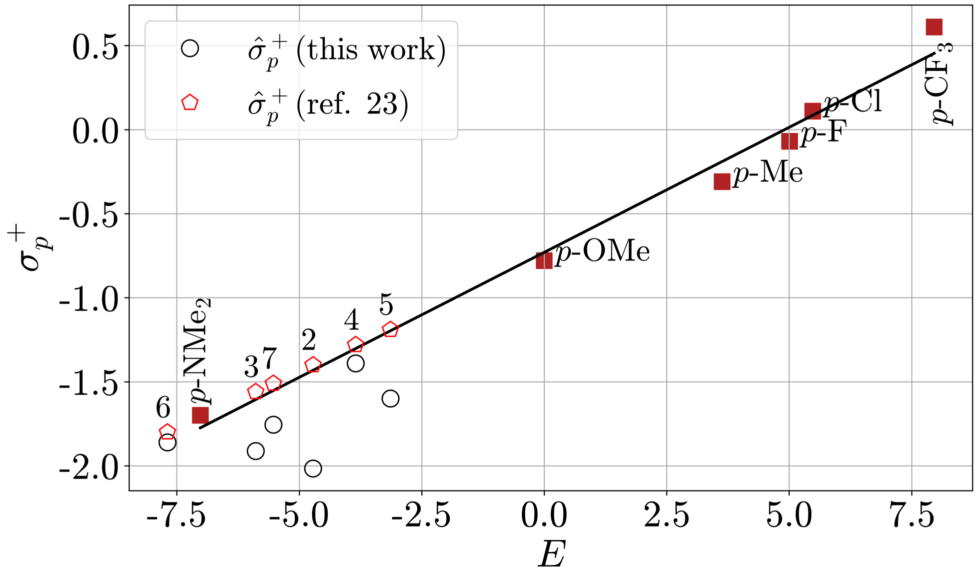

Figure S9: Correlation of Hammett–Brown σ_p^+ parameters with experimental E parameters for symmetrical *para*-substituted benzhydrylium ions. The deviation for electron-donating groups can be traced back to the prediction of LUMO energies (MLR in conjunction with C_{FG}). While the agreement is excellent for electron-withdrawing groups, the MLR model overestimates the LUMO energy of electron donors.

Table S8: Newly predicted Hammett–Brown σ_p^+ parameters for *para*-substituents present in the data set, based on the linear correlation shown in Fig. S8.

ID	Substituent	MLR coefficient	$\hat{\sigma}_p^+$ value
1	*p*-OPh	0.019	−0.92
2	*p*-dpa	0.040	−2.02
3	*p*-mpa	0.038	−1.91
4	*p*-mfa	0.028	−1.39
5	*p*-pfa	0.032	−1.60
6	*p*-pyr	0.037	−1.86
7	*p*-mor	0.035	−1.76

References

(1) Proppe, J. Quantitative structure–reactivity relationships for synthesis planning: The benzhydrylium case. `https://git.rz.tu-bs.de/proppe-group/qsrr-benzhydrylium`, last accessed on 26 October 2023.

(2) Mayer, R. J.; Hampel, N.; Mayer, P.; Ofial, A. R.; Mayr, H. Synthesis, Structure, and Properties of Amino-Substituted Benzhydrylium Ions - A Link between Ordinary Carbocations and Neutral Electrophiles: Synthesis, Structure, and Properties of Amino-Substituted Benzhydrylium Ions - A Link between Ordinary Carbocations and Neutral Electrophiles. *Eur. J. Org. Chem.* **2019**, *2019*, 412–421.

(3) Neese, F. The ORCA Program System. *Wiley Interdiscip. Rev. Comput. Mol. Sci.* **2012**, *2*, 73–78.

(4) Neese, F. Software Update: The ORCA Program System, Version 4.0. *Wiley Interdiscip. Rev. Comput. Mol. Sci.* **2018**, *8*, e1327.

(5) Tao, J.; Perdew, J. P.; Staroverov, V. N.; Scuseria, G. E. Climbing the Density Functional Ladder: Nonempirical Meta–Generalized Gradient Approximation Designed for Molecules and Solids. *Phys. Rev. Lett.* **2003**, *91*, 146401.

(6) Staroverov, V. N.; Scuseria, G. E.; Tao, J.; Perdew, J. P. Comparative Assessment of a New Nonempirical Density Functional: Molecules and Hydrogen-Bonded Complexes. *J. Chem. Phys.* **2003**, *119*, 12129–12137.

(7) Weigend, F.; Ahlrichs, R. Balanced Basis Sets of Split Valence, Triple Zeta Valence and Quadruple Zeta Valence Quality for H to Rn: Design and Assessment of Accuracy. *Phys. Chem. Chem. Phys.* **2005**, *7*, 3297–3305.

(8) Grimme, S.; Antony, J.; Ehrlich, S.; Krieg, H. A Consistent and Accurate Ab Ini-

tio Parametrization of Density Functional Dispersion Correction (DFT-D) for the 94 Elements H-Pu. *J. Chem. Phys.* **2010**, *132*, 154104.

(9) Grimme, S.; Ehrlich, S.; Goerigk, L. Effect of the Damping Function in Dispersion Corrected Density Functional Theory. *J. Comput. Chem.* **2011**, *32*, 1456–1465.

(10) Mayr, H.; Lakhdar, S.; Maji, B.; Ofial, A. R. A Quantitative Approach to Nucleophilic Organocatalysis. *Beilstein J. Org. Chem.* **2012**, *8*, 1458–1478.

(11) Mayr, H.; Ofial, A. R. A Quantitative Approach to Polar Organic Reactivity. *SAR QSAR Environ. Res.* **2015**, *26*, 619–646.

(12) Mayr, H.; Ofial, A. R. Mayr's Database of Reactivity Parameters. `https://www.cup.lmu.de/oc/mayr/reaktionsdatenbank2/`, last accessed on 27 October 2023.

(13) Hoffmann, G.; Balcilar, M.; Tognetti, V.; Héroux, P.; Gaüzère, B.; Adam, S.; Joubert, L. Predicting Experimental Electrophilicities from Quantum and Topological Descriptors: A Machine Learning Approach. *J. Comput. Chem.* **2020**, *41*, 2124–2136.

(14) Neese, F. Software Update: The ORCA Program System—Version 5.0. *Wiley Interdiscip. Rev. Comput. Mol. Sci.* **2022**, *12*, e1606.

(15) Becke, A. D. Density-functional Thermochemistry. III. The Role of Exact Exchange. *J. Chem. Phys.* **1993**, *98*, 5648–5652.

(16) Stephens, P. J.; Devlin, F. J.; Chabalowski, C. F.; Frisch, M. J. Ab Initio Calculation of Vibrational Absorption and Circular Dichroism Spectra Using Density Functional Force Fields. *J. Phys. Chem.* **1994**, *98*, 11623–11627.

(17) Himanen, L.; Jäger, M. O. J.; Morooka, E. V.; Federici Canova, F.; Ranawat, Y. S.; Gao, D. Z.; Rinke, P.; Foster, A. S. DScribe: Library of Descriptors for Machine Learning in Materials Science. *Comput Phys Commun* **2020**, *247*, 106949.

(18) Proppe, J.; Kircher, J. Uncertainty Quantification of Reactivity Scales. *ChemPhysChem* **2022**, *23*, e202200061.

(19) Mayr, H. Reactivity Scales for Quantifying Polar Organic Reactivity: The Benzhydrylium Methodology. *Tetrahedron* **2015**, *71*, 5095–5111.
